# Successful recovery without any neurological complication after intraoperative cardiopulmonary resuscitation for an extended period of time in the lateral position: a case report

**DOI:** 10.1186/s40981-016-0036-7

**Published:** 2016-06-02

**Authors:** Kazuma Yunoki, Ryo Sasaki, Akihisa Taguchi, Shun Maekawa, Hiroshi Ueta, Kazuo Yamazaki

**Affiliations:** 1 Department of Anesthesiology and Critical Care, Kobe City Medical Center General Hospital, 2-2-1, Minatojimaminamimachi, Chuo-ku, Kobe-city, Hyogo 650-0047 Japan; 2 Department of Anesthesiology, Hyogo Prefectural Amagasaki General Medical Center, 2-17-77, Higashinaniwacho, Amagasaki-city, Hyogo 660-8550 Japan

**Keywords:** Cardiopulmonary resuscitation, Lateral position, Chest compression, End-tidal CO2

## Abstract

No successful resuscitation has ever been reported about intraoperative cardiopulmonary resuscitation (CPR) for an extended period of time in the lateral position. Here we report a case of successful resuscitation without any neurological complication after cardiac arrest due to massive hemorrhage and 25 min of CPR in the lateral position.

The patient was a 65-year-old man. During open hemostasis for the postoperative hemorrhage, the patient’s rhythm changed sinus to ventricular fibrillation (VF), followed by asystole. We started CPR immediately with the patient in the left lateral position. Chest compression was performed by two practitioners, one pressing patient’s sternum and the other pressing simultaneously patient’s mid-thoracic spine from his back. During CPR, though the value of end-tidal CO2 (EtCO2) was significantly low (around 5–20 mmHg), the value of systolic arterial pressure was kept about 35–50 mmHg, and diastolic pressure about 20–30 mmHg. After the 25 min of lateral CPR, he achieved the return of spontaneous circulation (ROSC). He was hemodynamically stable after ROSC. He regained his consciousness at the next postoperative day. He was discharged from our hospital on the 60th day of operation without any cardiac and neurological complication.

Successful neurological outcome suggests that we may expect satisfactory neurological outcome even in the case of lateral position and prolonged CPR if we perform effective CPR with the feedback of arterial blood pressure and EtCO2 and with the immediate intervention to culprit injuries.

## Background

Cardiopulmonary arrest (CPA) is one of the most catastrophic events during surgeries and immediate initiation of cardiopulmonary resuscitation (CPR) is needed. CPR is usually performed with the patient in the supine position, but in some surgical situation, making a patient in the supine position is challenging and we have to decide starting CPR with the patient in the unusual position in such a case. There are a lot of reposts about prone CPR [1], but few reports are available about lateral CPR, and no successful resuscitation has ever been reported about intraoperative CPR for an extended period of time in the lateral position. Here we report a case of successful resuscitation without any neurological and cardiac complication after cardiac arrest due to massive hemorrhage and 25 min of CPR in the lateral position. Written informed consent was obtained from the patient for publication of this case report and accompanying images.

## Case presentation

The patient was a 65-year-old man (177 cm, 78 kg). He had a history of right upper lobectomy for adenocarcinoma in the right upper lobe 3 years prior. He was admitted to our hospital for VATS (Video Assisted Thoracic Surgery) S6 segmentectomy for the recurrence cancer. He had no other past history of note, and had no medication before hospitalization. He smoked 1 pack of cigarette per day for 45 years. Preoperative laboratory test showed no abnormality. Preoperative electrocardiogram showed arterial fibrillation for the first time, and intravenous unfractionated heparin infusion started after the hospitalization. Heparin infusion was stopped 6 h before operation.

VATS S6 segmentectomy was successfully performed with the patient in the left lateral position. Anesthesia was maintained with sevoflurane (1.0–1.5 %) and remifentanil with left one-lung ventilation. After the chest closure, the patient was repositioned to the supine and we stopped sevoflurane administration. Before emergence from anesthesia, massive bleeding started suddenly from the drainage tube placed in the right thorax. The patient’s systolic blood pressure decreased to 30 mmHg and end-tidal CO2 (EtCO2) also decreased from 40 to 10 mmHg. The patient was immediately placed in the head-down position, his head cooled with the ice pack for the cerebral protection, a hemodialysis catheter inserted from his right internal jugular vein for the purpose of rapid transfusion. After the rapid transfusion of crystalloids, hetastarch, and blood products along with inotrope infusions (dopamine and norepinephrine), his systolic blood pressure increased to 60 mmHg but he was hemodynamically unstable. Right pulmonary artery rupture was suspected. Surgeon decided there was urgent need to complete hemostasis and only in the lateral position should the culprit artery be accessible. Then the patient was placed in the left lateral position again for the open chest hemostasis. Left one-lung ventilation was started again, and then his right chest was re-opened.

**Fig. 1:**
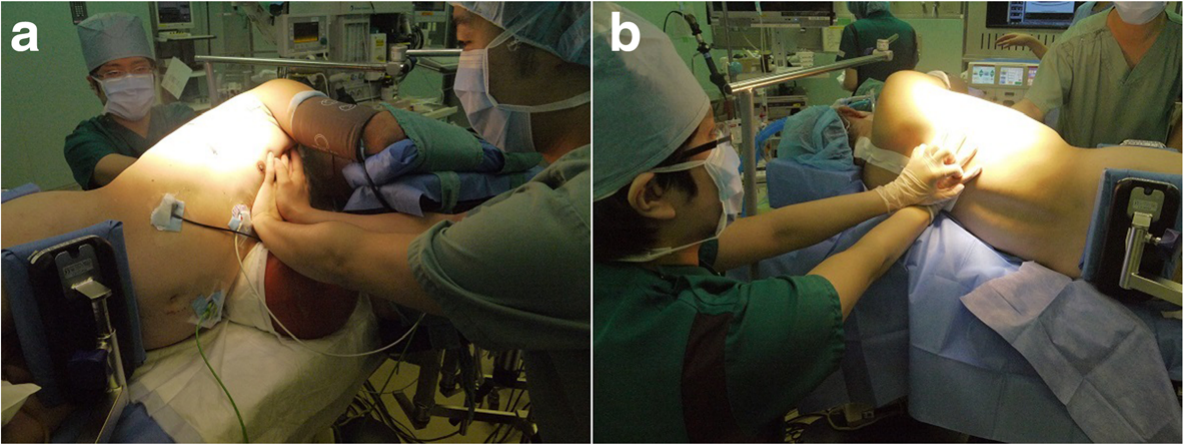
Reenactment of CPR in the lateral position. Picture (**a**) shows one surgeon standing in front of the patient and pushed the patient’s sternum. Picture (**b**) shows the other surgeon standing behind the patient and pushed his mid-thoracic spine. Chest compression was performed from both sides simultaneously at approximately 100 times per min. A patient is firmly fixed in the lateral position by the body-fixing devices and immediate transposition to supine is challenging

**Fig. 2:**
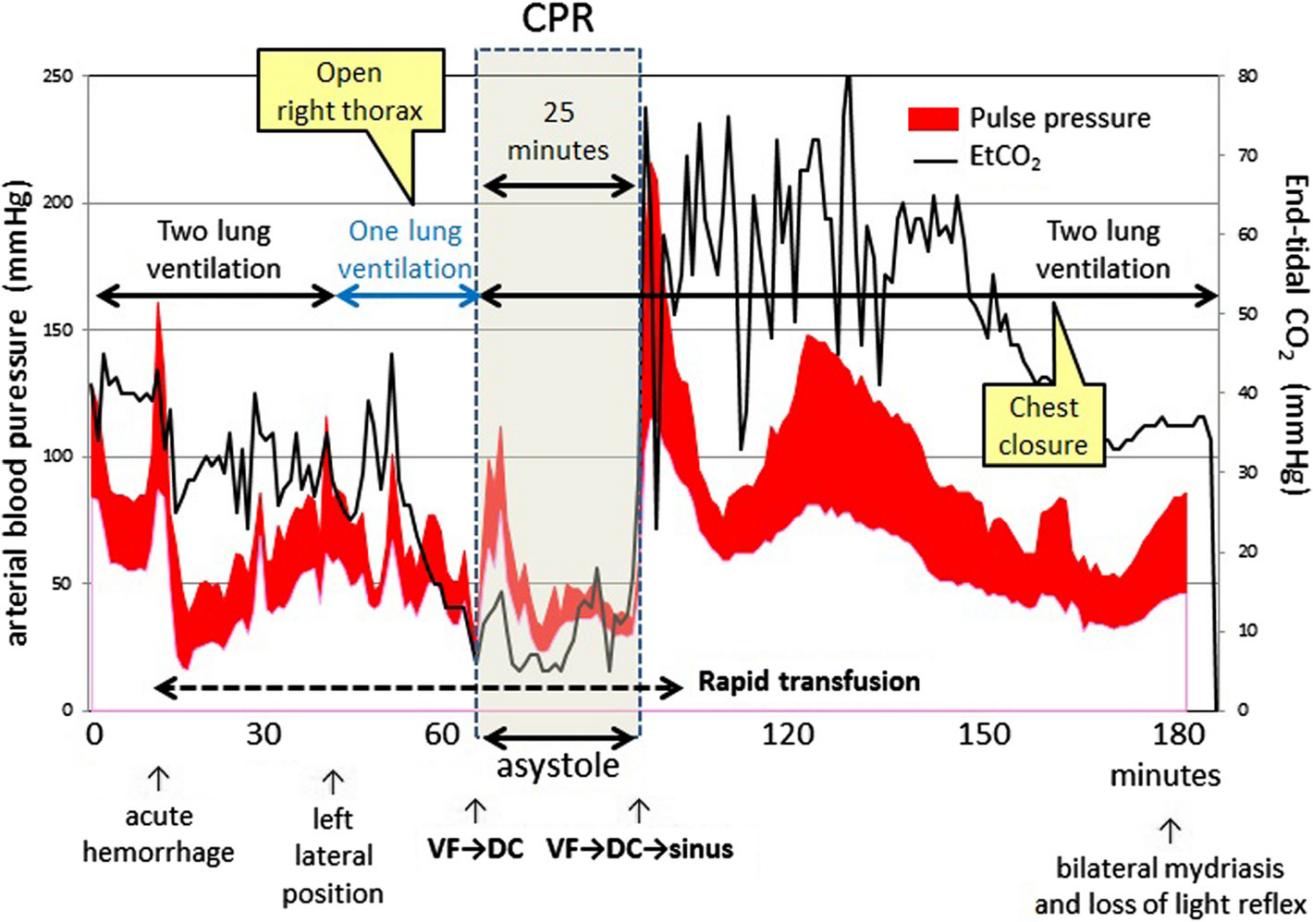
Overview of the resuscitation from the beginning of acute hemorrhage to the chest re-closure. CPR was performed for 25 min in the left lateral position and with the right thorax open

As soon as thoracotomy, his systolic blood pressure fell to 50 mmHg followed by the ventricular fibrillation (VF). The patient’s body temperature was 36.9 °C. Defibrillation (biphasic, 200J) was immediately delivered, but electrocardiogram (ECG) showed asystole and we started CPR. The patient was firmly fixed in the left lateral position and immediate conversion to the supine position for chest compression was impossible. Direct heart compression seemed infeasible because of firmly adhesive right lung and mediastinum. Extracorporeal CPR seemed also infeasible because of firmly fixed lateral positioning. So chest compression by two surgeons started with the patient in the lateral position (Fig. 1). One surgeon stood in front of the patient and placed his both palms on patient’s sternum, another surgeon placed his both palms on the patient’s mid-thoracic spine from his back, and chest compression was performed from both sides simultaneously at approximately 100 times per minute. During chest compression, the patient’s arterial pressure waveform appeared sinusoidal in tune with chest compression with systolic value about 35–50 mmHg, and diastolic value about 25–35 mmHg. EtCO2 was 5–20 mmHg. One-lung ventilation was returned to two-lung ventilation. Pulse check was performed every 2 min during CPR and ECG showed asystole every time, and then chest compression by two practitioners was restarted after intravenous bolus infusion of 1 mg epinephrine. The culprit of bleeding was right main pulmonary artery. We also used an auto-transfusion system so as to conserve blood transfusion. After the anastomosis and hemostasis of culprit right pulmonary artery, the waveforms of ECG changed from asystole to VF, and two times of defibrillation (biphasic, 200J) were delivered, which was effective. ECG waveform converted to sinus rhythm and the patient’s pulse became palpable, so we stopped CPR. CPR time was 25 min. During the reminder of the procedure the patient was hemodynamically stable under the high dose of inotrope infusion (epinephrine, norepinephrine, and dopamine). After the final check of hemostasis the patient’s chest was closed and the operation finished. The overview of resuscitation is shown in the Fig. 2. The patient received 2520 ml of packed red blood cells, 1200 ml of fresh frozen plasma, 200 ml of platelet and 2600 ml of intraoperative blood salvage, in addition to 5350 ml of fluid administration. At the end of surgery, the patient’s body temperature was 35.7 °C. He showed bilateral mydriasis and loss of light reflex.

The patient was transferred to ICU intubated and ventilated. All the sedatives were stopped for the neurological evaluation. Three hours after the operation, bilateral light reflex appeared. Approximately 6 h after the operation, the patient regained conscious and responded to verbal commands. No apparent limb paralysis was observed. The patient’s body temperature was kept around 36.0 °C. On the second postoperative day he was extubated. His Glasgow Coma Scale (GCS) score was E4V5M6 at the time of extubation. He was re-intubated on the 5th day of operation due to the deterioration of oxygenation, and underwent tracheostomy on the 7th day of operation. He was enrolled in a pulmonary rehabilitation program, and was successfully weaned from artificial ventilation on the 36th day of operation. His tracheostomy hole healed itself and closed during hospitalization. He was discharged from our hospital on the 60th day of operation without any cardiac and neurological complication. No head CT or MRI scan was performed after the surgery because the patient showed no neurological abnormality.

## Discussion

To prevent cardiac arrest of the patient in hemorrhagic shock, keeping cardiac output with fluid resuscitation is essential. The patient, however, manifested cardiac arrest despite various effort of fluid resuscitation. In this case, the culprit of bleeding was right pulmonary artery. Substantial amount of rapidly infused fluid administration via venous catheter might be lost as bleeding from injured pulmonary artery. Blood flow from right to left heart might be severely limited and, as a result, cardiac output of left heart might be severely decreased, resulting in cardiac arrest. This suggests immediate hemostasis of culprit injury is primary needed to make fluid resuscitation effective in the case of pulmonary artery injury. When the patient became shock, we once stabilized his circulation with rapid fluid administration and then decided starting hemostasis. More prompt start of hemostasis might prevent his cardiac arrest, but it is unknown whether we could prevent cardiac arrest in anyway.

Few reports are available for CPR performed on patients in the lateral position. To our knowledge, there are three case reports with successful neurological outcome after intraoperative CPR in the lateral position. CPR time described in those reports is less than 6 min. We performed 25 min of CPR in the lateral position, and this is the first report of CPR for an extended period of time in the lateral position with successful neurological outcome. Abraham et al. [2] describe the two thumb encircling technique in a 6 year old and achieve systolic blood pressures in the 50–60 mmHg. Bengali et al. [3] describe one resuscitator standing next to the patient and applying compressions by placing one hand on either side of the patient. They were able to achieve systolic blood pressures near 70 mmHg and EtCO2 above 20 mmHg. Takei et al. [4] describe two person lateral compressions and had systolic blood pressure of 70–80 mmHg. In our case, two rescuers performed chest compression, one rescuer pushing patient’s sternum and the other rescuer simultaneously pushing his mid-thoracic spine from his back at 100 times per minute as deeply as possible. We could perform this two-person lateral CPR and hemostasis procedure at the same time. Successful recovery without any neurological complication suggests effective CPR was performed in the lateral position.

As a factor of high-quality chest compression, 2010 American Heart Association (AHA) Guideline [5] recommends providing chest compressions of adequate rate and adequate depth, allowing complete chest recoil after each compression, and minimizing interruptions in compressions. Most of CPR are performed with the patient in the spine position, but in some surgical situation, making patient in the supine position is challenging. For example, most of thoracic surgeries are performed in the lateral position and immediate transposition from lateral to supine is difficult because of firmly fixing body-supporting devices, a lot of inserted catheters, and inability of gathering sufficient staff to make transposition. In addition, repositioning the patient may make it impossible for surgeon to access to the surgical site and address the cause of cardiovascular collapse. So in the case of intraoperative cardiac arrest in the special position, immediate initiation of CPR without repositioning is sometimes required to prevent the delay of resuscitation and minimize interruptions of compression.

To perform high-quality CPR, adequate monitoring and feedback is important, especially when CPR is delivered under unusual circumstance. Recent consensus [6] emphasizes the importance of monitoring a patient’s response to resuscitation. The consensus statement recommends keeping arterial diastolic pressure above 25 mmHg to maintain sufficient coronary perfusion pressure when an arterial catheter is available. The consensus statement also recommends keeping EtCO2 above 20 mmHg. While EtCO2 monitoring has some limitations, it is a good surrogate marker of pulmonary blood flow and cardiac output. In our case, an arterial catheter was inserted and the patient was intubated, so arterial pressure monitoring and capnography were available. We performed chest compression for 25 min in the lateral position, and the value of systolic arterial pressure was kept about 35–50 mmHg, diastolic pressure about 25–35 mmHg during CPR. This is well above the value of recommendation. In our case, however, the value of end-tidal CO2 was significantly lower (around 5–20 mmHg) during CPR. Decrease of pulmonary blood flow due to the massive hemorrhage and disturbed expansion of lung due to surgical manipulation disturbed pulmonary respiration and it was challenging to keep EtCO2 above 20 mmHg during CPR. In spite of low value of EtCO2 at the initiation of CPR, EtCO2 showed increasing trend during CPR along with the hemostasis procedure and rapid transfusion. This implies that EtCO2 can be the index of restoration of pulmonary blood flow and cardiac output.

In spite of low EtCO2 value during CPR, patient showed successful neurological and cardiac outcome. Firstly, the prevention of postoperative hyperthermia may be one of the factors that influenced this good outcome. Hyperthermia after cardiopulmonary resuscitation is reported to be a potential factor for an unfavorable functional neurologic recovery [7]. In our case, the patient was kept in normothermia and didn’t develop hyperthermia after resuscitation, which might help decrease brain damage and lead to successful neurological outcome. In addition, we cooled the patient’s head with the ice pack so as to decrease the oxygen consumption of the brain as soon as he manifested shock. We stopped sevoflurane administration during CPR, but remaining sevoflurane might suppress the oxygen demand of the brain and lead to cerebral protection. Whether these factors, however, had cerebral protective effects on the patient is still unknown.

How long CPR should be continued, or when to stop CPR may largely depend on the situation. We performed 25 min of CRP and got a successful cardiac and neurological outcome. This implies that even though prolonged CPR should be performed, we shouldn’t easily give up continuing CPR if the cause of CPA is accessible and effective CPR is performed.

## Conclusion

A patient undergoing thoracic surgery manifested cardiac arrest due to massive hemorrhage and 25 min of CPR was performed in the left lateral position. Simultaneous chest compression by two persons enabled the patient to keep his systolic arterial pressure about 35–50 mmHg, diastolic pressure about 25–35 mmHg, though the value of EtCO2 was temporarily low. Successful neurological outcome suggests that we may expect satisfactory neurological outcome even in the case of lateral position and prolonged CPR if we perform effective CPR with the feedback of arterial blood pressure and EtCO2 and with the immediate intervention to culprit injuries.

### Ethics approval and consent to participate

This is an anonymous case report. Ethical committee in hour hospital suggested that there is no need for getting an approval of ethical committee.

### Consent for publication

The patient has provided permission to publish these features of his case, and the identity of the patient has been protected.

### Availability of data and materials

This is a case report and there is no data set.

## Abbreviations

CPA: cardiopulmonary arrest
CPR: cardiopulmonary resuscitation
ECG: electrocardiogram
EtCO2: end-tidal CO2
ROSC: return of spontaneous circulation
VATS: video assisted thoracic surgery
VF: ventricular fibrillation
